# Suprapatellar nailing in complex Tibial Fractures

**DOI:** 10.1051/sicotj/2023025

**Published:** 2023-12-20

**Authors:** Wasudeo Gadegone, Piyush Gadegone, Vijayanand Lokhande

**Affiliations:** 1 MS (General surgery), MS (Orthopaedics) MNAMS (Orthopaedics) SICOT Fellow, Ex-Professor of Orthopaedic, Senior Consultant Orthopaedic Surgeon, Trauma and Orthopaedic Hospital Vivek Nagar Mul Road Chandrapur Maharashtra 442401 India; 2 MS (Orthopaedics) DNB (Orthopaedics), Consultant Orthopaedic Surgeon, Trauma and Orthopaedic Hospital Vivek Nagar Mul Road Chandrapur Maharashtra 442401 India; 3 MBBS, MS Orthopaedics, DNB Orthopaedics, Consultant Orthopaedic Surgeon, Trauma and Orthopaedic Hospital Vivek Nagar, Mul Road Chandrapur Maharashtra 442401 India

**Keywords:** Surapatellar approach, Semiextended position, Reamed nailing, Poller screws, Multilock nail

## Abstract

*Purpose*: This study evaluates the functional and radiological outcomes of segmental and comminuted tibial fractures using a suprapatellar approach in semi extended position. *Methods*: A total of 62 patients, with a mean age of 40.8 (range: 21–78 yrs) and 43 males and 19 females, were retrospectively evaluated between January 2017 and December 2022. Patients with comminated tibial fracture AO Type 42 C3 (32) and segmental AO Type 42 C2 (21) AO Type 42 C1 (9) were treated with a IMN using the suprapatellar technique. There were 51 closed fractures and 11 grade-one open fractures. All patients were subjected to the suprapatellar nailing technique in the semi-extended position. *Results*: Fracture healing 20.4 weeks on average was needed for the fracture to heal in segmental and 21.2 weeks in comminuted fracture. There were two instances of the delayed union in comminuted fractures. In two cases of segmental fracture dynamization is required. One case of non-union required bone grafting in a segmental fracture and in two instances in comminuted fractures to improve union after four months of monitoring. Primary union occurred in 56 patients. After minimum of 10.2 months of follow-up. According to Johner and Wruh’s criteria with modifications, out of 62 patients, 47 had excellent results, 10 had good results, and 5 had fair results. *Conclusions*: In segmental and comminuted fracture tibia, the suprapatellar IMN technique in a semi-extended position results in a significantly lower rate of malalignment, and good union because of soft tissue friendly bypass surgery.

## Introduction

An inferior radiological and functional outcome or revision surgery arise in the treatment of segmental and comminuted complex tibial fractures because of precarious blood supply of the intermediate fragment and the often severely damaged surrounding soft tissue causing impaired fracture healing [1–3].

The reduction and IM nail fixing procedures are more complex and difficult to accomplish in proximal tibial, segmental, and comminuted fractures. The management of complex tibial fractures and deforming stresses in proximal and distal tibial fractures by traditional infrapatellar techniques have been employed for intramedullary nailing, may have a chance of bad repositioning, insufficient reaming, difficult imaging technique and inadequate nail placement [4].

The disadvantages of infrapatellar nailing led to the development of a semi-extended nailing technique by Tornetta and Collins, who employed a medial parapatellar approach with lateral subluxation of the patella in 10° to 15° of knee flexion in proximal tibial fractures The justification for this entry was that the patellar tendon’s pull on the proximal fragment is abolished when the knee joint is bent to its maximum of 15 degrees, making it simple to realign and heal the fracture. Advanced surgical methods and implants are necessary to achieve the proper alignment and fixation. The indications for nailing proximal and distal tibial fractures with minimal intra-articular extension have been expanded with the knee in a semi-extended position has recently been advocated as a safe and effective surgical technique [5, 6].

Dean Cole was the first who advocated a suprapatellar approach using a midline quadriceps tendon insertion and a percutaneous lateral suprapatellar approach was described by Morandi et al. by a 1.5-cm transverse skin incision at the superolateral corner of the patella. This approach facilitates intramedullary nailing in the semi-extended knee and overcomes the problems of reduction in 90° flexion with subsequent malalignment of the fragments. Maintenance of fracture reduction and radiographic imaging is simplified with the semi-extended position. Sanders et al. operated with the suprapatellar approach and found in the majority no changes in cartilage damage [7, 8].

The objective of this retrospective study is to describe a minimally invasive suprapatellar approach in a semi-extended position that successfully addresses the challenges of reduction and stable IM fixation in segmental and comminuted fractures.

## Materials and methods

In our institute, we treated 62 patients with acute closed and grade one open segmental, and comminuted tibial fractures between January 2017 and December 2022. The series of 43 male and 19 female patients had an average age of 40.8 years (range, 21–78 years). Accidents, including motor vehicle (*n* = 38), height-related (*n* = 11), industrial (*n* = 7) and domestic (*n* = 6) falls, resulted in patients with comminuted tibial fracture AO Type 42 C3 (32) and segmental AO Type 42 C2 (21) AO Type 42 C1 (9). Other injuries, largely musculoskeletal in character, were sustained by five patients. Eleven segmental fractures were from distal diaphyso-metaphyseal (*n* = 13), and proximal-metaphyseal diaphysis (*n* = 8). About 51 closed and 11 were Grade I open fractures. Seven patients, three in the proximal section and four in the distal tibial segment had minimal intraarticular extension (Tables 1 and 2).

**Table 1:** Distribution of patients according to AO classification.

Fracture type	Fracture type	No. (%) of patients/62
AO Type C3	32	41 [51.6%]
AO Type C2	21	21 [33.8%]
AO Type C1	9	9 [14.6%]
Type C2	Distal diaphyseo metaphyseal	13
Type C2	Proximal Metophyseo diaphyseal	8

**Table 2:** Age distribution.

Age group	Number of patients	Male	Female	Total	%
		43	19	62	100%
20–30	9	7	2	9	17.75%
30–40	20	13	7	20	34.%
40–50	14	11	3	14	22.60%
50–60	13	9	4	13	14.51%
60–70	4	2	2	4	4.80%
70–80	2	1	1	2	1.62%

The semi-extended suprapatellar nailing technique was used on all patients. In fifteen cases, a temporary knee-spanning ex-fixator that lasted 10–15 days was used to cover the soft tissue problems before being nailed. About 47 patients had intramedullary nailing done within 3–7 days of the incident. Fibular fixation was done in 9 patients by nail in 7 and by plate 2. The clinical and radiological follow-up intervals were every 4–6 weeks up until 6 months, thereafter, these intervals were longer. Patients were kept under observation until the minimum follow-up of 10.2 months (range: 10–26 months)

*Inclusion criteria were as follows*:

Patients with segmental and comminuted fractures, Skeletal mature patients (more than 18 years old) and closed fractures and grade one open fractures with minimal intra-articular extension.

*Exclusion criteria were as follows*:

Patient with neuro-vascular Injuries, grades II and III compound fractures, pathological fractures and previous leg and skeletal injury.

### Surgical procedure

While the patient is lying supine on a radiolucent table, the injured leg is positioned with a roll behind the knee joint so that it is flexed at about a 15–20-degree angle. The wounded limb is subsequently draped and the C-arm is positioned to provide excellent imaging in both the lateral views. The saline is injected into the knee joint in the amount of 30 mL as a prophylactic precaution to evaluate the patellar ballotability prior to instrumentation.

Fracture reduction is possible with the aid of manual or calcaneal traction, manipulation, a distraction device, and a clamp. The fracture reduction is carried out and fastened with the suitable screw in intra-articular extension. Fibular fixation if needed is carried out in very distal fractures within 7–8 cm from the distal tibial plafond by closed intra medullary nail or by plate to restore length and to achieve stability. In unreducible fractures additional uncortical plates are used as reduction tools and kept as and when necessary .The 4 mm poller pin is passed in the proximal tibia 4–5 cm from the articular surface from lateral to medial, anterior two-third and posterior one-thirds junction to direct the guide wire into the centre of the canal, lessening the possibility that it will pierce the posterior cortex. A two- to three-centimetre longitudinal skin incision is made 1 cm above the superior pole of the patella. The quadriceps tendon is exposed by conducting a blunt dissection, and it is then divided longitudinally in the centre. In order to make it simpler to enter a finger beneath the patella and into the knee joint, curved artery forceps are used to break up adhesions and widen the wound.

A 3 mm stiff proximal tibial guide pin by “free-hand” is inserted through the protective guide sleeve onto the top of the tibia. The entrance point is anterior to the anterior articular margin in the lateral view and medial to the lateral tibial spine in the AP view. Both lateral and anteroposterior radiographic images are used to confirm the precise position of the guide wire. Now, through the sleeve over the guide pin, 9 mm reamer is used to widen the medullary canal in the proximal tibia to a depth of 4–6 cm and finally reamed with a self-stopper proximal reamer up to 13 mm. The 2.5 mm guide wire is exchanged and passed into the medullary canal through the fracture level, and descended to the distal tibia up to the epiphyseal line. This was done after reduction and clamping the fracture. The proximal fracture is treated first, then the middle fragment is stabilised with a pointed clam, and finally, the distal fracture is reduced. The wire must be centred in the distal portion in both the lateral and anteroposterior views. For suprapatellar nailing, long reamers up to 56 cm are required to reach the distal placement site. By forcing the reamer through the comminuted zone, caution is exercised in comminuted fractures.

**Figure 1:**
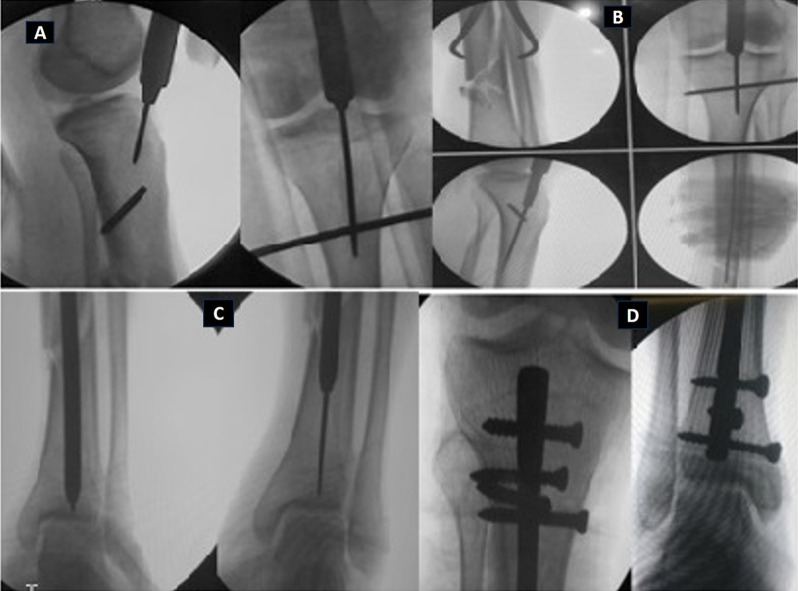
(A) Entry point medial to lateral tibial spine, (B) clamp assisted reduction and guide wire, (C) distal placement of guide wire and reaming and (D) proximal and distal placement of nail.

Segmental fractures must be manually clamped and stabilised to prevent spinning while reaming. Blocking screws are frequently needed anteroposterior or medial to lateral to nail penetration, depending on how the fracture is structured. This can improve the fracture’s stability by realigning it. In the majority of cases, the nail’s length was chosen so that it would extend to within 1 cm of the distal joint line and 5 mm below the joint line at the nail’s proximal end in the lateral view of the anteroposterior view (Figure 1).

The nail is subsequently fixed proximally by the system’s targeting mechanism and distally by freehand technique, and under fluoroscopy, it is ensured that it does not protrude into the knee joint in either plane. Saline is pumped through the knee joint to ensure that all debris and blood have been removed. The wound site is meticulously cleansed before being patched. The wound is neatly bandaged.

## Results

**Figure 2:**
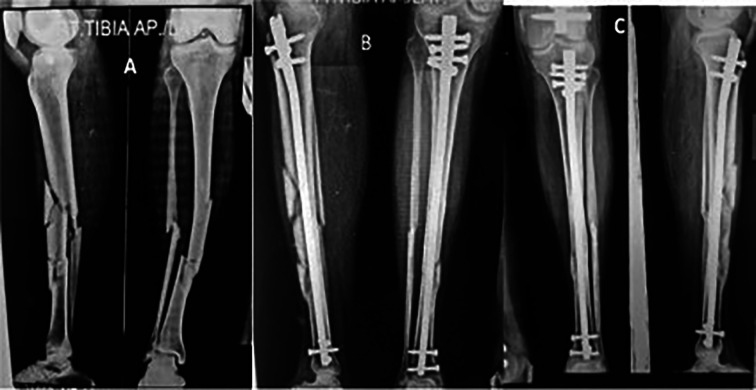
(A) Segmental comminuted fracture tibia, (B) post op x-ray with suprapatellar nail and (C) nine months follow up. Union of fracture.

**Figure 3:**
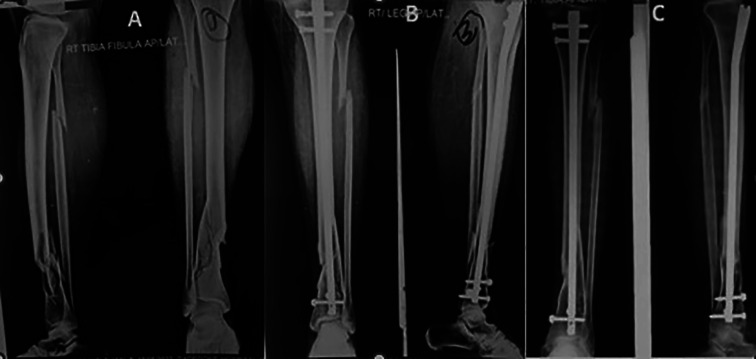
(A) Comminuted fracture distal tibia, (B) post op x-ray with suprapatellar nail and (C) six months follow up. Union of fracture.

**Figure 4:**
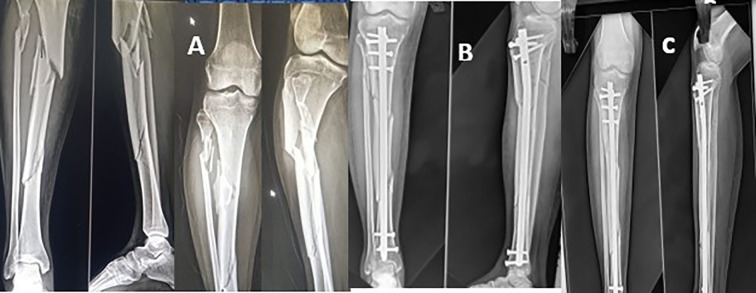
(A) Segmental fracture l tibia, (B) post op x-ray with suprapatellar nail and (C) six months follow up. Union of fracture.

Radiographic evidence of callus in anteroposterior and lateral views of two or more cortices and the absence of pain at the fracture site were regarded as signs that the fracture had healed. The median time until the union of fracture was 20.4 weeks (range: 16–38 weeks). The median time for the proximal and distal fracture lines to converge in segmental fractures was an average of 18.6 and 21 weeks respectively. The distal portion of the diaphysis is where fractures that took longer to heal than proximal fragments were found. The average time to union for comminuted fractures was 21.2 weeks (range: 16–38 weeks) (Figures 2–4).

Two cases of segmental fractures had delayed union at the distal end that healed following dynamization and a proximal segment nonunion had bone grafting with an additional plate was required for one patient. Four months after index surgery, two patients with comminuted fractures required bone grafting to hasten recovery. In five instances, a superficial infection developed, with three cases at the distal locking point and one case at the proximal section at the screw insertion site. With the aid of antibiotics and wound debridement, they were effectively treated. In 46 cases, the return of a functional range of motion in the knee and ankle was fully assessed at the most recent clinical visit after 12 months of follow-up, and in five cases, it was greater than 90 degrees relative to the uninjured side. Ankle joint involvement in a distal comminuted fracture was seen earlier in the follow-up period, along with pain and loss of ankle motion. Four individuals exhibited an average loss of ankle dorsiflexion and plantar flexion of 6–8 degrees. The following criteria were used to determine malalignment: >5 procurvatum/or recurvatum, >10 rotation, or >10 procurus/valgus deformity. In five cases, the fracture healed with valgus angulation between 6 and 9 degrees (mean, 6), and in two cases, the fracture healed with a varus angulation between 5 and 7 degree, two in segmental and 5 in comminuted fracture. The patients showed up for the routine clinical radiological assessment, and the malalignment was within physiological limits. Five patients experienced anterior knee pain, which in three cases went away after further examination. A reduction of length average of 5 mm range 3–8 mm was in 9 cases. At one-year follow-up visits, nonunion of the fibula in two patients was discovered, although there was no functional issue for the patients. One patient experienced clinical complications from compartment syndrome, including impaired ankle and foot motion. The primary union rate in 58 patients after ten months of follow-up was 93.55%. The functional and radiological outcomes of the procedure were evaluated prospectively at minimum of 10.2 months following the operation using According to Johner and Wruhs [9] criteria with modification, out of 62 patients, 47 [75.8%] had excellent results, 10 [16.2%] had good results, and 5 [8%] had fair results (Table 3).

**Table 3:** Johner, R. and Wruhs, O. criteria.

	No. (%) of patients IM nail
47 excellent	
10 Good	16.2%
5 Fair	8%
0 Poor	0%
Total 62 patients	100%

## Discussion

The Segmental, comminuted diaphyseal fractures of the tibia are difficult to treat due to high-velocity injury zone and likely reduced in bone vitality and problems with alignment, restoration of length, stabilization and union [10]**.**

Diaphyseal tibia fractures now typically require intramedullary nailing, either by infrapatellar or suprapatellar approach. Indications are now extended for segmental, extreme proximal and distal extraarticular fractures with or without comminution. The widely accepted treatment method for proximal tibial diaphyseal fractures is suprapatellar nailing and performed in a semi-extended position, which was believed to improve fracture alignment not only of proximal fractures but also of more-distal injuries [11, 12].

In our retrospective study of 62 patients, 21 segmental and 41 comminuted tibial fractures, we used nailing by the suprapatellar technique with adjuvant distractor, percutaneous clamps, provisional plating, blocking screws and fibular fixation as and when required, are very important tools to attain reduction and restoration of length and to centralize the guide wire specially in distal tibial comminuted fracture and segmental fractures as they are often required as suggested by various authors apart from the semi-extended nailing as in proximal tibial fractures [13, 14].

The nail insertion portal is very critical in proximal fractures as compared with in distal fractures but guidewire placement should be deep and central on both AP and lateral views distally. The main advantage of a semi-extended strategy for segmental and proximal and distal comminuted fractures is the ease of imaging and avoiding excessive manipulation of the limb in the setting of a tenuous clamp application. Particularly relevant to the distal tibia is the opportunity to reduce and fix the fibula. This can be especially helpful in valgus fracture patterns and in the setting of simple fibula fractures where accurate restoration of the length of the lateral column will help to combat valgus and guide placement, appropriate restoration of length and rotation of the tibia [15–17].

We resorted to fibular fixation in length stable transverse and short oblique fracture by a nail in seven cases and in comminuted fibula by a plate in two cases. We are of the opinion that very distal unstable comminuted fractures require fibular fixation to restore the length and improve the stability of the lateral column. Simple articular fracture extensions into either the knee or ankle joints are not contraindications to medullary nailing [18–20]. Seven patients in our series were included with minimal intra-articular extension which was successfully addressed by articular reduction and screw fixation in five cases and proximal plate fixation in two cases. When addressing these fractures, it is critical to adhere to the principles of articular fracture management, which dictate that the articular component be addressed with anatomic reduction and compression followed by restoring the mechanical axis of the limb followed by intramedullary nailing.

In our series the median time until the union of fracture was 20.4 weeks (range 16–38 weeks). The median time for the proximal and distal fracture lines to converge in segmental fractures was an average of 18.6 and 21 weeks respectively. The distal portion of the diaphysis is where fractures that took longer to heal than proximal fragments were found. The average time to union for comminuted fractures was 21.2 weeks (range: 16–38 weeks) which is comparable to the reported by other authors the period of union 15–43 week range for segmental tibial fracture union periods. Some of the reported series on a population that took a long time to heal and their study also contained a significant number of open fractures. Open fractures tend to heal more slowly than closed fractures, which may contribute to the explanation of some of the observed discrepancies in healing times [21, 22].

We have included very few cases of grade one open fractures but there was no influence on healing time, hence in particular, we observed quicker union times for segmental fractures treated with gently reamed IMN with a suprapatellar approach, indicating that this technique may be preferable for treating segmental tibial and comminuted fractures.

We also saw some challenges with the healing procedure. There were delayed and nonunions in three of the segmental tibial fracture cases, necessitating dynamization and two bone grafts – one at the distal end and the other at the proximal end. The reported nonunion rates in segmental tibial fractures vary substantially. There were two cases out of the 41 cases of comminuted tibial fractures. Although nonunion rates of up to one-third in patients and required more reoperations than the other groups, which is likely related to the segmental fractures’ higher frequency of problems with bone healing and incidence of septic sequelae as reported by authors [22, 23].

The reduced rate of malalignment was observed in the suprapatellar approach. It can also improve outcomes in these complex fracture patterns by demonstrating that suprapatellar nailing caused malalignment that exceeded 5° in just 4.3% of patients which is comparable to the results reported in the literature. Because of its simplicity in placing and fluoroscopy, suprapatellar nailing appears to be becoming more and more popular. Our research demonstrates the distinctiveness and complexity of segmental and comminuted tibial fractures and reduced anterior knee pain by supra patellar approach as only five patients complained of anterior knee pain in our series [24–26].

In a cadaveric investigation, Gaines et al. [27] compared the suprapatellar and traditional parapatellar approaches and found that the rates of intraarticular injuries were equal. Using an entry site through a safe zone medial to lateral to the tibial spine reduces the risk of anterior cruciate ligament injury [28, 29].

The limitations of our study populations are small sample size and it is not a comparative study. We have not studied the effect on cartage damage but no patients complained of knee pain and septic arthritis in our series.

We advise using IMNs to treat these fractures and believe it is crucial to properly educate orthopaedic and trauma surgeons about the use of suprapatellar nails in the challenging healing of segmental and comminuted fractures of the tibia. The functional outcomes of the knee joint and ankle are equivalent to those obtained with the infrapatellar nailing approach. However, additional studies are needed, particularly randomised studies comparing it with the previous conventional methods.

## Conclusions

Suprapatellar nailing in segmental and comminuted tibia fractures is a safer approach. The suprapatellar IMN technique produces excellent radiological and functional results in terms of much-decreased rates of misalignment, and ease of operation.

## References

[1] Wu CC, Shih CH (1993) Segmental tibial shaft fractures treated with interlocking nailing. J Orthop Trauma 7(4), 468–472. 8229384 10.1097/00005131-199310000-00010

[2] Rommens PM, Coosemans W, Broos PL (1989) The difficult healing of segmental fractures of the tibial shaft. Arch Orthop Trauma Surg 108(4), 238–242. 2774877 10.1007/BF00936208

[3] Woll TS, Duwelius PJ (1992) The segmental tibial fracture. Clin Orthop Relat Res 281, 204–207. 1499212

[4] Yoon RS, Bible J, Marcus MS, Donegan DJ, Bergmann KA, Siebler JC, Mir HR, Liporace FA (2015) Outcomes following combined intramedullary nail and plate fixation for complex tibia fractures: A multi-centre study. Injury 46(6), 1097–1101. 25843886 10.1016/j.injury.2015.03.019

[5] Tornetta P III, Collins E (1996) Semiextended position of intramedullary nailing of the proximal tibia. Clin Orthop Relat Res 328, 185–189. 10.1097/00003086-199607000-00029 8653954

[6] Tijs Jakma PR, Rajmohan Rai (2011) Insertion of intramedullary nails from the suprapatellar pouch for proximal tibial shaft fractures: A technical note. Acta Orthop Belg 77, 834–837. 22308632

[7] Morandi M, Banka T, Gaiarsa GP, et al. (2010) Intramedullary nailing of tibial fractures: Review of surgical techniques and description of a percutaneous lateral suprapatellar approach. Orthopedics 33, 172–179. 20205366 10.3928/01477447-20100129-22

[8] Sanders RW, Dipasquale TG, Jordan CJ, Arrington JA, Sagi HC (2014) Semi-extended intramedullary nailing of the tibia using a supra-patellar approach: Radiographic results and clinical outcomes at a minimum of 12 months follow-up. J Orthop Trauma 28(5), 245–255. 24694557 10.1097/BOT.0000000000000082

[9] Johner R, Wruhs O (1983) Classification of tibial shaft fractures and correlation with results after rigid internal fixation. Clin Orthop Relat Res 178, 7–25. 6883870

[10] Krettek C, Miclau T, Grün O, Schandelmaier P, Tscherne H (1998) Intraoperative control of axes, rotation and length in femoral and tibial fractures: Technical note. Injury 29(Suppl 3), C29–C39. 10341895 10.1016/s0020-1383(98)95006-9

[11] Avilucea FR, Triantafillou K, Whiting PS, Perez EA, Mir HR (2016) Suprapatellar intramedullary nail technique lowers rate of malalignment of distal tibia fractures. J Orthop Trauma 30, 557–560. 27218695 10.1097/BOT.0000000000000631

[12] Eastman J, Tseng S, Lo E, Li CS, Yoo B, Lee M (2010) Retropatellar technique for intramedullary nailing of proximal tibia fractures: A cadaveric assessment. J Orthop Trauma 24(11), 672–676. 20926965 10.1097/BOT.0b013e3181c1d675

[13] Kim KC, Lee JK, Hwang DS, Yang JY, Kim YM (2007) Provisional unicortical plating with reamed intramedullary nailing in segmental tibial fractures involving the high proximal metaphysis. Orthopedics 30(3), 189–192. 17375542 10.3928/01477447-20070301-10

[14] Krettek C, Miclau T, Schandelmaier P, Stephan C, Mohlmann U, Tscherne H (1999) The mechanical effect of blocking screws (“Poller screws”) in stabilizing tibia fractures with short proximal or distal fragments after insertion of small-diameter intramedullary nails. J Orthop Trauma 13(8), 550–553. 10714781 10.1097/00005131-199911000-00006

[15] Egol KA, Weisz R, Hiebert R, Tejwani NC, Koval KJ, Sanders RW (2006) Does fibular plating improve alignment after intramedullary nailing of distal metaphyseal tibia fractures? J Orthop Trauma 20(2), 94–103. 16462561 10.1097/01.bot.0000199118.61229.70

[16] Prasad M, Yadav S, Sud A, Arora NC, Kumar N, Singh S (2013) Assessment of the role of fibular fixation in distal-third tibia-fibula fractures and its significance in decreasing malrotation and malalignment. Injury 44(12), 1885–1891. 24074830 10.1016/j.injury.2013.08.028

[17] Stewart CM, Kiner D, Nowotarski P (2013) Intramedullary nail fixation of fibular fractures associated with tibial shaft and pilon fractures. J Orthop Trauma 27(5), e114–e117. 23609789 10.1097/BOT.0b013e3182694a2d

[18] Nork SE, Schwartz AK, Agel J, Holt SK, Schrick JL, Winquist RA (2005) Intramedullary nailing of distal metaphyseal tibial fractures. J Bone Joint Surg Am 87(6), 1213–1221. 15930529 10.2106/JBJS.C.01135

[19] Kubiak EN, Camuso MR, Barei DP, Nork SE (2008) Operative treatment of ipsilateral noncontiguous unicondylar tibial plateau and shaft fractures: Combining plates and nails. J Orthop Trauma 22(8), 560–565. 18758288 10.1097/BOT.0b013e318185fa7e

[20] Dombroski D, Scolaro JA, Pulos N, Beingessner DM, Dunbar R, Mehta S (2012) Fibular fracture stabilization with a guidewire as supplementary fixation in tibia fractures. Am J Orthop (Belle Mead NJ) 41(11), 506–509. 23431514

[21] Giannoudis PV, Hinsche AF, Cohen A, MacDonald DA, Matthews SJ, Smith RM (2003) Segmental tibial fractures: An assessment of procedures in 27 cases. Injury 34, 756–762. 14519356 10.1016/s0020-1383(02)00393-5

[22] Huang CK, Chen WM, Chen TH, Lo WH (1997) Segmental tibial fractures treated with interlocking nails: A retrospective study of 33 cases. Acta Orthop Scand 68, 563–566. 9462357 10.3109/17453679708999027

[23] Beardi J, Hessmann M, Hansen M, Rommens PM (2008) Operative treatment of tibial shaft fractures: A comparison of different methods of primary stabilisation. Arch Orthop Trauma Surg 128, 709–715. 18465138 10.1007/s00402-008-0619-5

[24] Bonnevialle P, Cariven P, Bonnevialle N, Mansat P, Martinel V, Verhaeghe L, Mansat M (2003) Segmental tibia fractures: a critical retrospective analysis of 49 cases. Rev Chir Orthop Reparatrice Appar Mot 89, 423–432. 13679742

[25] Franke JJ, Hohendorff B, Alt V, Thormann U, Schnettler R (2016) Suprapatellar nailing of tibial fractures–Indications and technique. Injury 47(2), 495–501. 26553427 10.1016/j.injury.2015.10.023

[26] Sanders RW, Dipasquale TG, Jordan CJ, Arrington JA, Sagi HC (2014) Semi-extended intramedullary nailing of the tibia using a supra-patellar approach: Radiographic results and clinical outcomes at a minimum of 12 months follow-up. J Orthop Trauma 28(5), 245–255. 24694557 10.1097/BOT.0000000000000082

[27] Gelbke MK, Coombs D, Powell S, DiPasquale TG (2010) Suprapatellar versus infrapatellar intramedullary nail insertion of the tibia: A cadaveric model for comparison of patellofemoral contact pressures and forces. J Orthop Trauma 24(11), 665–671. 20926959 10.1097/BOT.0b013e3181f6c001

[28] Gaines RJ, Rockwood J, Garland J, Ellingson C, Demaio M (2013) Comparison of insertional trauma between suprapatellar and infrapatellar portals for tibial nailing. Orthopedics 36(9), e1155–e1158. 24025006 10.3928/01477447-20130821-17

[29] Giannoudis PV, Court-Brown C, Katsoulis E (2006) After intramedullary nailing of the knee, the cause of anterior knee discomfort in the tibia and femur. J Bone Joint Surg Br 88, 576–580. 16645100 10.1302/0301-620X.88B5.16875

